# Wearable electronics

**DOI:** 10.1093/nsr/nwac193

**Published:** 2022-09-21

**Authors:** Huisheng Peng

**Affiliations:** State Key Laboratory of Molecular Engineering of Polymers and Department of Macromolecular Science, Fudan University, China

Wearable electronics has been booming as an important multidisciplinary field across chemistry, physics, materials science and engineering, electrical engineering and biomedical engineering in the recent decade. Wearable electronic devices were previously as rigid as the other portable electronic devices at the early stage and they had been simply made lightweight enough to be handled by hands or the other parts of our bodies. People then realized that, since wearable electronic devices are mainly in contact with our bodies, they should be flexible to match the skin to make us safer and more comfortable. To this end, on the one hand, a variety of soft polymers have been explored as electrodes and active layers to produce flexible electronic devices for wearables; on the other hand, rigid materials can be designed into stretchable architectures such as wavy structures to produce the desired flexibility. A key point is to make them as thin as possible for the best flexibility under both situations. In other words, wearable electronic devices appearing in thin films is one mainstream direction.

Inspired by the successful strategy to make high-performing wearables by decreasing their sizes in one dimension, people have further started to reduce their sizes in two dimensions, which thus produces fiber-shaped electronic devices with many unique and promising advantages. For instance, they can be woven into highly flexible and breathable textiles that can most efficiently adapt to our bodies. These electronic textiles look like conventional clothes and can be even washed for hundreds of cycles, which represents another main direction particularly in the recent decade.

Both thin-film and fiber-shaped electronic devices have been extensively investigated for wearables from viewpoints of the synthesis of materials, design of structures, optimization of interfaces and improvement of properties, all of which will be further emphasized from both academy and industry in the future. In addition, a lot of effort will be also made to achieve real applications. Several wearable electronic products such as textile displays do appear in the current market and solve important problems that remained challenges before. It is thus necessary to summarize the current main achievements and highlight the key directions to guide future study on wearable electronics.

Here I am glad to announce this exciting Special Topic contributed from leading scientists in wearable electronics across the world. We have collected a total of 10 papers including 4 perspective articles, 1 research article, 4 review articles and 1 interview article. Specifically, the first two perspective articles, one from Professor Joseph Wang and the other from Professors Ye Zhang and Peter G. Bruce, highlight the next-generation power systems for wearables. The third and fourth perspective articles from Professor Zhenan Bao and myself focus on the future direction for wearable display technologies. The research article from Professors Xiang Zhou and Zunfeng Liu reports an effective strategy for artificial muscle materials. The recent advances in triboelectric nanogenerators as wearable power sources and self-powered sensors have been carefully reviewed by Professor Zhong Lin Wang. A booming direction on interfacial binding strategies for stretchable electronic components is summarized by Professor Xiaodong Chen. The following review article from Professor Wei Yan summarizes the development of thermally drawn fibers and devices for smart textiles. For the last review article from Professors Cheng-Wei Qiu and Guangming Tao, the main achievements in radiation-cooling materials are deeply analysed. Finally, the interview article assembled by Professor Enming Song shows us deep insights into epidermal electronics from Professor John A. Rogers.

Although this Special Topic cannot cover all wearable studies, it in a way reflects representative directions in wearable electronics. I sincerely hope that it will inspire readers from multidisciplinary fields. I also hope that it will attract more interest from the industry to speed up the application process of wearable devices.

This Special Topic is greatly indebted to all contributing authors for the wonderful papers. I am also grateful to the editors of *National Science Review* for enthusiastic support. Finally, my warm appreciation is offered to the whole editorial team of the Journal for their great work.

